# Whole-genome sequencing to identify rare variants in East Asian patients with dementia with Lewy bodies

**DOI:** 10.1038/s41514-024-00180-2

**Published:** 2024-11-21

**Authors:** Tetsuaki Kimura, Kosuke Fujita, Takashi Sakurai, Shumpei Niida, Kouichi Ozaki, Daichi Shigemizu

**Affiliations:** 1 Medical Genome Center, Research Institute, National Center for Geriatrics and Gerontology, Obu, Aichi Japan; 2 Department of Prevention and Care Science, Research Institute, National Center for Geriatrics and Gerontology, Obu, Aichi Japan; 3 Research Institute, National Center for Geriatrics and Gerontology, Obu, Aichi Japan; 4 RIKEN Center for Integrative Medical Sciences, Yokohama, Kanagawa Japan; 5 Department of Cardiovascular Medicine, Hiroshima University Graduate School of Biomedical and Health Sciences, Hiroshima, Japan

**Keywords:** Genome, Neuroscience

## Abstract

Dementia with Lewy bodies (DLB) is the second most common form of age-related dementia, following Alzheimer’s disease (AD). DLB is associated with a worse prognosis than AD and is characterized by a more rapid progression of cognitive impairment and a poorer quality of life. In addition, the pathogenesis of DLB is less understood than that of AD, and only three genes—*SNCA* (α‐synuclein), *APOE* (apolipoprotein E), and *GBA1* (glucosylceramidase beta 1)—have been convincingly demonstrated to be associated with DLB. In this study, we utilized whole-genome sequencing data from 1744 Japanese individuals, comprising 45 DLB patients and 1699 cognitively normal older adults, aiming to identify new genes associated with DLB. Our genome-wide association studies of genes with potentially deleterious mutations identified the *CDH23* gene as being associated with DLB, reaching a Bonferroni-corrected significance (*P* = 7.43 × 10^−4^). The gene contained three ethnicity-specific heterozygous missense variants (rs181275139, rs563688802, and rs137937502). *CDH23* has been linked to deafness syndromes, and DLB patients carrying these mutations displayed symptoms of subjective hearing loss, suggesting a potential association between DLB onset and auditory impairment. Additionally, we explored human leukocyte antigen (HLA) genotypes associated with DLB but found no significant associations. This result suggests that the pathology of DLB differs from that of Parkinson’s disease, which has been reported to have an association with HLA. Although a limitation of this study is the lack of replication of our findings, which require further validation in independent cohorts, our study enhances the understanding of the etiology of DLB in the Japanese population and provides new insights into the underlying mechanisms of its pathogenesis.

## Introduction

Dementia with Lewy bodies (DLB) is the second most common type of dementia in people over 65 years old, following Alzheimer’s disease (AD)^1^. DLB is associated with a worse prognosis than AD and is characterized by a faster decline in cognitive impairment and a lower quality of life^2^. DLB patients present with cognitive, neuropsychiatric, sleep, and autonomic symptoms^3^. The core clinical features of DLB include cognitive fluctuation, visual hallucinations, parkinsonism, and rapid eye movement (REM) sleep behavior disorder (RBD). Diagnostic biomarkers such as reduced dopamine transporter uptake in the basal ganglia observed through single-photon emission computed tomography (SPECT), decreased uptake on metaiodobenzylguanidine (MIBG) myocardial scintigraphy, and confirmation of REM sleep without atonia via polysomnography confirm the diagnosis^4^.

Treatments for DLB are currently limited to managing symptoms, as no curative treatments are available. Cholinesterase inhibitors have demonstrated efficacy in improving cognitive function in DLB patients. However, the use of antipsychotic agents, typically prescribed for other forms of dementia, can pose risks due to potential severe hypersensitivity reactions in DLB patients^3^. Furthermore, there is a significant difference between the number of cases clinically diagnosed and those diagnosed by neuropathology postmortem^2^. Therefore, achieving effective treatments for DLB requires a deeper understanding of the underlying pathological mechanisms.

In DLB, the brain develops numerous neuronal inclusions known as Lewy bodies. These structures primarily consist of α-synuclein, and neurons where they accumulate eventually undergo cell death and are lost^2^. Brain atrophy in DLB is less pronounced than in AD, likely due to the loss of specific neurons such as dopaminergic neurons in the substantia nigra and cholinergic neurons in the basal forebrain^2^. However, the molecular mechanisms underlying the selective loss of neurons in DLB remain unclear.

To uncover the genetic factors contributing to DLB, researchers have conducted genome-wide association studies using common variants. These studies have identified several genes associated with DLB, including *SNCA*, which encodes α-synuclein; *APOE*, which is involved in lipid metabolism and is an AD risk factor; and *GBA1*, which is involved in glycolipid metabolism^5,6^. Moreover, analysis using rare variants has recently revealed associations of *MFSD3* and *MLPL43* with DLB in the Japanese population^7^. However, despite these discoveries, the molecular mechanisms underlying DLB remain unclear. Further identification of genetic factors will play a key role in elucidating the pathogenesis of DLB.

Here, we explored genes newly associated with DLB by using whole-genome sequencing data from a large cohort of Japanese individuals. We identified three candidate ethnicity-specific missense mutations in the *CDH23* gene. DLB patients with these mutations displayed symptoms of hearing loss, suggesting a potential association between DLB onset and auditory impairment. Additionally, we investigated human leukocyte antigen (HLA) genotypes associated with DLB but found no significant associations. Our study contributes to the understanding of the etiology of DLB in the Japanese population and provides new insights into the underlying mechanisms of its pathogenesis.

## Results

### WGS of Japanese individuals

Blood samples from 1744 Japanese individuals ≥65 years old were obtained from the National Center for Geriatrics and Gerontology (NCGG) Biobank. Of these, 45 were from patients with DLB and 1699 from cognitively normal older adults (CN). We performed WGS analysis of the 1744 samples by using the Illumina HiSeq X Ten and NovaSeq 6000 platforms. On average, 364 million read pairs were obtained from the analysis, of which 98.3% were mapped to the human reference genome (GRCh37) and 7.5% were removed as PCR duplicates. We called genetic variants (single-nucleotide variants: SNVs, short insertions and deletions: Indels) by using GATK HaplotypCaller and GenotypeGVCFs tools^8^. A total of 12,395,511 genetic variants from 1664 samples (45 DLBs and 1619 CNs) passed stringent quality control (QC) criteria for both genotypes and samples (see Materials and Methods). The average ages of the individuals from the DLB and CN samples were 78.7 years (SD = 5.9 years) and 76.7 years (SD = 3.9 years), respectively; the *APOE* ε4 allele frequencies were 0.26 and 0.093, respectively; and the female-to-male ratios were both 1.14:1 (Supplementary Table 1).

### Novel DLB pathogenic genes

**Fig. 1:**
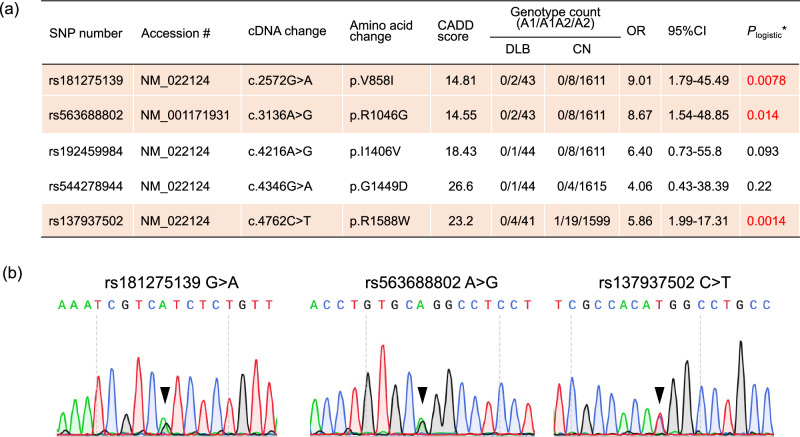
Three rare variants in the *CDH23* gene associated with DLB. Three rare variants in *CDH23* were associated with DLB and confirmed by Sanger sequencing. **a** Gene-based association testing for rare coding variants identified five candidate pathogenic mutations in the *CDH23* gene. Red text and orange highlighting indicate statistical significance. OR indicates odds ratio, and CI indicates confidence interval. *P_logistic_ was obtained with a logistic regression model adjusted by age and sex. Minor and major alleles were labeled as A1 and A2. **b** Three missense variants (rs181275139, rs563688802, and rs137937502) detected with WGS were validated by using Sanger sequencing. Triangles indicate the validated variant positions.

Genome-wide association studies with small sample sizes face challenges in detecting variants with statistical significance due to limited study power, especially after Bonferroni correction for multiple testing. We examined association signals within know DLB genes—*SNCA*, *APOE*, and *GBA1*—but did not observe any associations with these genes in our WGS data. To address this limitation, we investigated the aggregation of candidate pathogenic variants within genes to identify novel disease-associated genes. Of the 12,395,511 variants obtained, 101,311 were found in protein-coding regions. These were classified into 48,731 (48.8%) common variants and 52,580 (51.2%) rare variants in accordance with the definition of the ToMMo 54KJ database of healthy Japanese individuals^9^ (Table 1, see Materials and Methods). We functionally annotated the rare coding variants. Of the 52,580 variants, 466 were assigned to frameshift deletion; 244 to frameshift insertion; 460 to nonframeshift deletion; 169 to nonframeshift insertion; 361 to splicing variant; 541 to stop-gain; 38 to stop-loss; 19,890 to synonymous SNV; and 30,411 to nonsynonymous SNV. We conducted genome-wide gene-based rare variant association testing by using the optimal sequencing kernel association test (SKAT-O), focusing on potentially deleterious mutations (frameshift deletion/insertion; splicing variants; stop-gain/stop-loss; nonsynonymous SNV) with CADD Phred-scaled scores of at least 10. A total of 872 genes containing at least these two rare variants were examined. The *CDH23* gene reached a Bonferroni-corrected level of significance (corrected *P* = 7.43 × 10^−4^, Supplementary Table 2). Five rare variants were involved in the association. Three of them (rs181275139, rs563688802, and rs137937502) showed DLB associations with *P* < 0.05 by a logistic regression model adjusted for age and sex (Fig. 1a). Although these risk alleles were observed in 8 DLB and 36 CN samples, no individuals carried two risk alleles. All of these rare variants in DLB WGS were validated by using Sanger sequencing (Fig. 1b).

**Table 1:** Classification of variants from WGS

Type	Common		Rare		Total	
	(0.01 ≤ MAF)		(MAF < 0.01)			
	Count	%	Count	%	Count	%
Non-Coding	7,358,181	59.9	4,936,019	40.1	12,294,200	100.0
Coding	48,731	48.8	52,580	51.2	101,311	100.0
Total	7,406,912	59.7	4,988,599	40.3	12,395,511	100.0

*MAF* minor allele frequency.

### Allele frequencies of *CDH23* variants in several populations

We investigated the population differences of the three variants on *CDH23* (rs181275139, rs563688802, and rs137937502) by using several public databases. The minor allele frequency (MAF) of rs181275139 in the East Asian population ranged from 0.002 to 0.004 in various databases, that of rs563688802 ranged from 0.001 to 0.003, and that of rs137937502 ranged from 0.003 to 0.005 (Table 2). Because these variants were rarely observed in other populations, including European, American, and African populations (Table 2), our findings indicated the presence of East Asian–specific DLB variants.

**Table 2:** Minor allele frequency of three *CDH23* variants in different populations

Data set	Population (sample size)	SNP number		
		rs181275139	rs563688802	rs137937502
NCGG Biobank	Japanese DLB (45)	0.022	0.022	0.044
	Japanese CN (1619)	0.0025	0.0025	0.0065
ToMMo54KJ	Japanese	0.0018	0.0029	0.0052
KRGDB	Korean	0.0038	0.0021	0.001
1000 Genomes 30×	East Asian	0.0026	0.0017	0.0034
	European	0	0	0
	American	0	0	0
	African	0	0	0
gnomAD v4.0.0	East Asian	0.0033	0.0011	0.0036
	South Asian	0.000022	0.000011	0.00039
	Admixed American	0.00032	0	0.0003
	African/African American	0.000067	0	0.00004
	European (non-Finnish)	0.000031	0	0.000053
	European (Finnish)	0	0	0
GenomeAsia 100 K	Asian	0.0084	NA	0.00035
	American	0	NA	0
	Oceanian	0	NA	0
	African	0	NA	0
	West Eurasian	0	NA	0

*CN* cognitively normal older adults, *NA* not applicable.

### The relationship between core features and *CDH23* variants

The diagnosis of DLB relies on the presence of four core features: fluctuating cognition, visual hallucinations, parkinsonism, and RBD. Among our 45 DLB patients, 25 had one core feature, 16 had two, and 4 had three. Visual hallucinations were the most frequently observed (*n* = 27, 60.0%), followed by parkinsonism (*n* = 20, 55.6%), fluctuating cognition (*n* = 13, 28.9%), and RBD (*n* = 9, 20.0%) (Supplementary Table 3). In the 8 DLB patients with variants in any of the three *CDH23* locations (rs181275139, rs563688802, and rs137937502), visual hallucinations were present in 62.5% (*n* = 5), Parkinsonism in 37.5% (*n* = 3), fluctuation in 37.5% (*n* = 3), and RBD in 12.5% (*n* = 1), (Fig. 1a and Supplementary Table 4). No statistically significant associations were observed between core features and *CDH23* variants (Fisher’s exact test *P* > 0.99 for visual hallucination, parkinsonism, and RBD; *P* = 0.69 for fluctuation).

### *CDH23* variants in patients with subjective hearing loss

Age-related hearing loss is common in DLB^10^. Mutations in *CDH23* are known as genetic causes of Usher syndrome type 1D (USH1D) and non-syndromic autosomal recessive deafness (DNFB12)^11,12^. We therefore assessed differences in hearing loss between DLB and CN using a data set of 113 individuals (41 DLB and 72 CN) in which the absence or presence of subjective hearing loss had been reported. Of the 41 DLB patients, 27 experienced subjective hearing loss, and 45 of 72 CNs had subjective hearing loss. No statistically significant difference in subjective hearing loss between the DLB and CN groups was observed (Fisher’s exact test *P* = 0.44).

We proceeded to explore the relationship between *CHD23* variants and subjective hearing loss. As one of eight DLB patients with *CDH23* variants did not provide information regarding subjective hearing loss, we analyzed this relationship in the remaining seven patients. Among the 7 DLB patients with *CDH23* variants, all experienced subjective hearing loss, whereas only 20 out of 34 DLB patients without *CDH23* variants had subjective hearing loss. A statistically significant association was found between the *CDH23* variants and hearing loss (one-sided Fisher’s exact test *P* = 0.04, Table 3). In non-DLB subjects, there was no statistically significant association between the *CDH23* variants and subjective hearing loss (Fisher’s exact test *P* = 0.88, Table 3). These results suggest that these *CDH23* variants could influence DLB pathogenesis through hearing loss.

**Table 3:** Relationship between CDH23 variants and hearing loss in DLB and non-DLB groups

Group	*CDH23* Variant	Subjective hearing loss		P*
		Positive	Negative	
DLB	W/	7	0	0.04
	W/O	20	14	
Non-DLB	W/	3	6	0.88
	W/O	124	140	

*one-sided Fisher’s exact test.

*W/* with, *W/O* without.

### Associations of HLA alleles

**Fig. 2:**
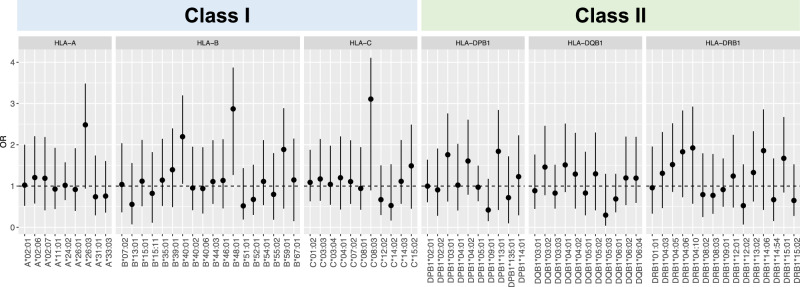
Associations of HLA class I and class II alleles between DLB and CN. HLA class I (**A**–**C**) and class II (DPB1, DQB1, and DRB1) alleles were obtained. The associations of high-resolution four-digit HLA alleles were assessed by using logistic regression with adjustment for sex and age. A false discovery rate (FDR) was calculated by using the Benjamini–Hochberg method. At an FDR-adjusted threshold of *P* < 0.05, no statistically significant alleles were found. Error bars represent 95% confidence intervals, and OR indicates odds ratio.

In DLB, there is postmortem evidence not only of an increase in cortical recruitment of T lymphocytes to brain tissue, but also of an early inflammatory response in the blood^13^. The HLA system plays a key role in regulating inflammatory responses. To examine the relationship between HLA alleles and DLB, we conducted HLA genotyping from WGS data by using HISAT-genotype software. A total of 240 four-digit HLA alleles for HLA class I (A, B, and C) and class II (DRB1, DQB1, and DPB1) were detected. Among these alleles, 74 were classified as common alleles with allele frequency ≥0.01 (HLA-A = 9 alleles, HLA-B = 18 alleles, HLA-C = 11 alleles, HLA-DRB1 = 15 alleles, HLA-DQB1 = 11 alleles, and HLA-DPB1 = 10 alleles). Associations of these common alleles with DLB were assessed by using logistic regression, adjusting for age, sex, and the number of *APOE4* alleles. However, none of these alleles demonstrated a statistically significant difference in allele frequency between DLB and CN subjects (Fig. 2).

## Discussion

In this study, the diagnosis of DLB was based not only on the presence of four core features, but was also confirmed with dopamine transporter–SPECT or MIBG scintigraphy. These imaging techniques have high accuracy in diagnosing the typical clinical syndrome in DLB, thereby ensuring reliable diagnosis of DLB. Using WGS in CN individuals and those with probable DLB, we comprehensively investigated pathogenic variants associated with DLB. Although their associations did not reach GWAS significance due to our small sample size, gene-based association testing for rare coding variants identified three candidate pathogenic mutations in the *CDH23* gene. The findings are specific to East Asians and generally absent from other populations. Our study provided a new perspective for understanding the pathogenesis of DLB.

Previously, the largest DLB GWAS have identified several risk loci^14^. Two of these loci, *APOE* and *BIN1*, are known as risk loci for AD^15^, while three—*SNCA*, *GBA1*, and *TMEM175—*have been found in Parkinson’s disease (PD) GWAS^16^. This suggests that these three age-related neurodegenerative diseases share overlapping disease-associated pathways. In this analysis, none of those previously identified GWAS genes were detected. We excluded singletons from SKAT-O because they are often the result of sequencing errors^17^. This exclusion may have led to missing some GWAS genes due to the removal of singular pathogenic variants. While this exclusion might have influenced the overall results, it is notable that we detected *CDH23*, a causal gene for age-related hearing loss, which could have important implications for DLB pathogenesis. Age-related hearing loss is associated with an increased risk of developing dementia in older adults^18^. Especially age-related hearing loss is associated with Lewy body pathology^10^.

A limitation of this study is that our results have not been replicated, and there is a possibility that they are incidental due to the small sample size of DLB patients. We examined the candidate pathogenic mutations in *CDH23* using variants from a European population, as reported by Chia et al. ^14^. We confirmed that our findings were not observed in the European data, supporting the idea that our results are specific to East Asian populations. Additionally, we attempted to validate our findings using variants from an Asian PD population, as reported by Oh et al. ^19^. However, the data only included common variants and did not include rare variants identified in our study. Therefore, we further investigated our findings in 30 Japanese patients with PD or PD dementia (PDD) from our NCGG biobank, but these variants were not observed in the PD or PDD patients. Since our findings were identified from WGS association studies with a small sample size, replication studies using an independent Japanese DLB cohort will be necessary to validate these findings in the future.

The *CDH23* gene has been identified as one of the causative genes of the USH1D and DNFB12 syndromes, which are characterized in part by hearing loss^11,12^. Two of three candidate mutations detected (rs181275139 and rs137937502) have been reported to cause a broad range of hearing loss in Chinese and Japanese populations when present in compound heterozygous or homozygous arrangements^20–23^. Although the remaining candidate mutation (rs563688802) has not been reported to be associated with congenital hearing loss, the two DLB patients with the mutation in our study did develop hearing loss. Given that hearing loss and auditory dysfunction are common in PD and DLB^24^ and that our DLB patients carried heterozygous candidate mutations, these candidate mutations could potentially induce hearing loss in aging, contributing to the development of DLB.

In mice and zebrafish, the genes homologous to *CDH23* show strong expression in specific regions of the brain^25,26^. Notably, *Cdh23* is strongly expressed in the zona incerta and subthalamic nucleus of mice. The zona incerta is a brain circuit underlying curiosity^27^, and the subthalamic nucleus is involved in the cortico-striato-thalamo-cortical motor loops and is implicated in cognitive and affective pathways^28^. Therefore, *CDH23* likely plays crucial roles in brain function, and its deficiency might contribute to the development of DLB.

Postmortem studies of DLB brains have revealed increased cortical recruitment of T lymphocytes^13^, suggesting the involvement of adaptive immunity in DLB pathogenesis. Additionally, PD, which like DLB is an α-synucleinopathy, has been reported to have HLA alleles associated with its pathogenesis^29–31^. Therefore, we examined associations between HLA alleles and DLB; however, our analysis did not reveal any significant associations. PDD is distinguished from DLB by the 12-month interval between the onset of PD and the onset of dementia. There is debate over whether PDD and DLB are distinct diseases or part of the same spectrum^32^. Further research using larger sample sizes and including cases of PDD is necessary to ascertain whether the current findings show potential differences in pathophysiology between PDD and DLB.

In summary, we identified three *CDH23* candidate mutations specific to East Asian populations that are associated with DLB. Although exactly how these mutations contribute to DLB development remains unclear, DLB patients harboring these mutations commonly exhibit hearing loss. This suggests the existence of a subtype of DLB that develops through hearing loss. Although further validation and investigation (e.g. structural variants) will be necessary to fully understand the role of these candidate mutations in the onset of DLB using a large number of DLB patients, we believe that our study enhances the understanding of the etiology of DLB in the Japanese population and provides new insights into the underlying mechanisms of its pathogenesis.

## Materials and Methods

### Ethics statements

This study was approved by the ethics committee of the NCGG. The design and performance of the current study involving human subjects were clearly described in a research protocol. All participation was voluntary, and participants completed informed consent in writing before registering with the NCGG Biobank.

### Clinical samples

All 1816 blood samples were obtained from Japanese individuals ≥65 years old, and the associated clinical data were obtained from the NCGG Biobank and the Center for Comprehensive Care and Research on Memory Disorders, NCGG database. Of the samples, 45 were from patients with DLB, 201 from patients with AD, and 1699 from CN subjects. DLB was diagnosed based on the criteria of the fourth report of the DLB Consortium^4^. Reduced dopamine transporter uptake on SPECT imaging or abnormal MIBG myocardial scintigraphy was confirmed in all subjects with DLB included in this study.

### WGS data analysis

DNA concentration was measured using Picogreen, and fragmentation of DNA was assessed with agarose gel electrophoresis. High-quality DNA was used for DNA libraries. The WGS library was constructed by using the TruSeq DNA PCR-Free Library Preparation Kit in accordance with the manufacturer’s instructions. WGS was conducted at Macrogen Japan Corporation, Takara Bio Inc, and GENEWIZ Inc. DNA was sequenced using the Illumina HiSeq X Ten or NovaSeq 6000 platforms with paired-end reads of 151 bp in accordance with the manufacturer’s instructions.

Read sequences were mapped to the human reference genome (GRCh37) with BWA-MEM (version 0.7.15)^33^. Duplicate PCR reads were identified and removed by using picard (version 2.21.4)^34^. Variant calling was conducted using the Genome Analysis Toolkit (GATK: version 4.1.0.0)^35^. Individual variant calling was performed using GATK HaplotypeCaller. The multi-sample individual variants were jointly called with in-house data using GenotypeGVCFs. Variant quality score recalibration was applied in accordance with the GATK Best Practice recommendations^8^. We filtered out SNVs that satisfied the following criteria: (1) Depth (DP) <10, (2) GenotypeQuality (GQ) <20, (3) Quality by Depth (QD) <2, QUAL <30, StrandOddsRatio (SOR) >4, FisherStrand (FS) >60, RMSMappingQuality (MQ) <40, MappingQualityRankSumTest (MQRankSum) <−12.5, ReadPosRankSumTest <−8, and (4) ExcessHet >20, and filtered out short Indels with (1) DP <10, (2) GQ <20, (3) QD <2, QUAL <30, FS >200, SOR >10, ReadPosRankSumTest <−20, and (4) ExcessHet >20.

Quality control of those variants was performed by using PLINK software^36^. We first applied QC filters to the subjects: (1) sex inconsistencies (--check-sex), (2) PI_HAT >0.25, where PI_HAT is a statistic for the proportion of identity by descent (IBD) (--genome), (3) genotype missingness (--mind 0.05), and (4) exclusion of outliers from the clusters of East Asian populations in a principal component analysis that was conducted together with 1000 Genomes Phase 3 data. We next applied QC filters to the variants: (1) genotyping efficiency or call rate (--geno 0.05), (2) minor count (--mac 2), and (3) Hardy–Weinberg equilibrium (--hwe 1$\times {10}^{-5}$). Note that singletons were excluded as they are usually the result of sequencing errors^17^.

### Variants annotation and variant-based or gene-based association tests

Functional annotations of the variants were performed using ANNOVAR (version 20191024). We used protein-coding variants with frameshift insertion/deletion, nonframeshift insertion/deletion, stop-gain/-loss, nonsynonymous SNV, synonymous SNV, and splicing variants. Variant frequency data were available from a public database from 54,000 healthy Japanese individuals, called ToMMo 54KJPN^9^. On the basis of the MAF, all variants were classified into two categories: rare (MAF <0.01) and common (0.01 ≤MAF). Genome-wide association studies of rare variants were conducted with the logistic regression with adjustment for sex and age, using PLINK software (--logistic).

Gene-based association tests of rare variants, SKAT-O^37^, were implemented in the R package SKAT (R Development Core Team, http://www.r-project.org/). Genes with Bonferroni-corrected *P*_bon_ < 0.05 were considered to be significantly enriched.

### HLA genotyping from WGS data

WGS-based HLA genotyping was conducted by using the HISAT-genotype software^38^, which is available in a public GitHub repository (1.3.2 release; https://daehwankimlab.github.io/hisat-genotype/). Individual HLA genotyping was performed by using the two programs “HISAT-genotype” and “HISAT-genotype_toolkit”, from which HLA class I (A, B, and C) and class II (DRB1, DQB1, and DPB1) alleles were obtained. Case-control association studies were conducted by using the high resolution four-digit HLA alleles. The associations were assessed by using the logistic regression analysis with adjustment for sex and age. Adjusted *P* values was calculated with the Benjamini–Hochberg method. An adjusted *P* value of 0.05 or less was considered statistically significant. The logistic regression analysis (--logistic) and Hardy–Weinberg equilibrium test (--hardy) of HLA loci were conducted with PLINK software (version 1.90b)^36^.

### Subjective hearing loss

Hearing loss was assessed during a medical interview conducted by a research nurse. As part of their initial hospital visit, all patients underwent a comprehensive geriatric assessment, which included sociodemographic inquiries, medical interview, and physical and cognitive examinations. In this study, patients who reported experiencing hearing difficulty were classified as having hearing loss.

## Supplementary information

Supplementatl Tables

## Data Availability

All datasets used or analyzed in the current study are available from the corresponding author on reasonable request.

## Code availability

All datasets used or analyzed in the current study are available from the corresponding author on reasonable request. The main code used for gene-based rare variant association testing is publicly available at https://github.com/NCGG-MGC/GeneBasedAssocTest.
