# Supplementary material for: Whole-genome sequencing to identify rare variants in East Asian patients with dementia with Lewy bodies
Source: NPJ Aging. 2024 Nov 21;10(1):52. doi: 10.1038/s41514-024-00180-2 (PMC11582613; doi:10.1038/s41514-024-00180-2)
Supplement: Supplementary file 1 — Supplementatl Tables [file 41514_2024_180_MOESM1_ESM.pdf]

Table S1. Characteristics of DLB patients and CN samples

Phenotype	DLB	CN
Sample Number	45	1619
Avg. of age (SD)	78.67 (5.87)	76.73 (3.89)
Female (%)	24 (53.33)	863 (53.24)
APOE4 genotype	0=26, 1=15, 2=4	0=1327, 1=282, 2=10
%*APOE4*	25.56	9.33

Abbreviations: DLB, dementia with Lewy bodies; CN, cognitively normal controls; SD, standard deviation

Table S2. Results of gene-based rare variant association tests

Number	Gene	*P*-value	Corrected *P*-value*	Number	Gene	*P*-value	Corrected *P*-value*
1	*CDH23*	$8.52×10^{-7}$	$7.43×10^{-4}$	437	*PLA2R1*	0.083	1
2	*PLBD2*	0.00017	0.15	438	*FCRL3*	0.083	1
3	*TRIM58*	0.00026	0.23	439	*METTL7B*	0.083	1
4	*ZFYVE28*	0.00048	0.42	440	*PRRC2C*	0.083	1
5	*MYO5B*	0.00059	0.51	441	*IFT140*	0.083	1
6	*NCOR2*	0.00067	0.58	442	*DPYSL4*	0.084	1
7	*LMF2*	0.00068	0.60	443	*SRCAP*	0.084	1
8	*PXDNL*	0.00074	0.64	444	*OCM2*	0.084	1
9	*TRIM71*	0.00074	0.65	445	*RASA1*	0.084	1
10	*USH2A*	0.00092	0.80	446	*LRIG3*	0.084	1
11	*TRIM37*	0.00098	0.85	447	*C20orf194*	0.084	1
12	*PCDHGA7*	0.0010	0.88	448	*NUDT22*	0.085	1
13	*PHKB*	0.0010	0.89	449	*SIM1*	0.085	1
14	*TRPS1*	0.0011	0.97	450	*ERBB2*	0.086	1
15	*FZD10*	0.0013	1	451	*BRWD1*	0.086	1
16	*USP34*	0.0014	1	452	*NNT*	0.086	1
17	*ADAMTSL1*	0.0017	1	453	*HAS1*	0.086	1
18	*KIF15*	0.0017	1	454	*ACLY*	0.087	1
19	*TTN*	0.0017	1	455	*DNAH11*	0.087	1
20	*PALMD*	0.0018	1	456	*SLC35G5*	0.088	1
21	*UNC80*	0.0020	1	457	*UTP20*	0.088	1

22	*COL11A1*	0.0020	1	458	*PLEKHH3*	0.088	1
23	*PREX2*	0.0021	1	459	*BSN*	0.088	1
24	*FRMD6*	0.0021	1	460	*CHIA*	0.089	1
25	*CEP164*	0.0022	1	461	*KIDINS220*	0.089	1
26	*MCM2*	0.0022	1	462	*GANAB*	0.090	1
27	*APOE*	0.0023	1	463	*CDK5RAP2*	0.090	1
28	*ANKRD26*	0.0026	1	464	*SMYD1*	0.090	1
29	*RGS12*	0.0028	1	465	*SUGP2*	0.090	1
30	*MYBBP1A*	0.0030	1	466	*SLC35A1*	0.090	1
31	*LRIG1*	0.0030	1	467	*SLAMF8*	0.091	1
32	*ITGA6*	0.0030	1	468	*NOC3L*	0.091	1
33	*NEB*	0.0031	1	469	*MB21D2*	0.091	1
34	*CNGA4*	0.0039	1	470	*FBXW8*	0.092	1
35	*OBSCN*	0.0040	1	471	*DAPK2*	0.092	1
36	*NHSL1*	0.0041	1	472	*DCHS2*	0.092	1
37	*ABCA7*	0.0045	1	473	*ITGA7*	0.092	1
38	*CD22*	0.0046	1	474	*GEMIN4*	0.093	1
39	*SYNE2*	0.0047	1	475	*SPTB*	0.093	1
40	*EPS8*	0.0047	1	476	*PLXNB1*	0.093	1
41	*HEATR1*	0.0049	1	477	*PCDHGA6*	0.093	1
42	*CELSR1*	0.0050	1	478	*CACNB3*	0.093	1
43	*TRPM8*	0.0051	1	479	*LOXL1*	0.094	1
44	*DOT1L*	0.0052	1	480	*KIAA0825*	0.095	1
45	*RHBDL3*	0.0052	1	481	*FAT1*	0.095	1

46	*RAD54B*	0.0055	1	482	*RAB44*	0.095	1
47	*ZNF846*	0.0058	1	483	*ADAMTS1*	0.095	1
48	*TEP1*	0.0059	1	484	*CPS1*	0.095	1
49	*PTPN21*	0.0061	1	485	*DDR1*	0.096	1
50	*LAMA5*	0.0064	1	486	*CAGE1*	0.096	1
51	*TTC28*	0.0065	1	487	*MYPN*	0.097	1
52	*SORL1*	0.0066	1	488	*TRPM1*	0.097	1
53	*FANCI*	0.0068	1	489	*DOCK8*	0.097	1
54	*EHMT1*	0.0068	1	490	*UMODL1*	0.097	1
55	*MSLN*	0.0069	1	491	*LTBP2*	0.098	1
56	*PXN*	0.0069	1	492	*LMO7*	0.098	1
57	*PCDHA10*	0.0069	1	493	*DNAH9*	0.099	1
58	*FLG*	0.0071	1	494	*RP1L1*	0.099	1
59	*CPOX*	0.0073	1	495	*IL17REL*	0.099	1
60	*KRT77*	0.0077	1	496	*SLC35B2*	0.099	1
61	*FRY*	0.0077	1	497	*PARD3*	0.099	1
62	*TIMELESS*	0.0080	1	498	*ANKRD55*	0.10	1
63	*MRPS18B*	0.0083	1	499	*MPDZ*	0.10	1
64	*NDUFA9*	0.0084	1	500	*CAPN12*	0.10	1
65	*ST6GALNAC5*	0.0086	1	501	*CUX2*	0.10	1
66	*NRXN3*	0.0086	1	502	*GABRR2*	0.10	1
67	*ZNF804A*	0.0087	1	503	*CDHR2*	0.10	1
68	*SIGLEC1*	0.0089	1	504	*SEC24B*	0.10	1
69	*MUC5B*	0.0092	1	505	*TUBE1*	0.10	1

70	*WDFY3*	0.0093	1	506	*MTHFSD*	0.10	1
71	*EFCAB7*	0.0094	1	507	*TRRAP*	0.10	1
72	*DNAH5*	0.0096	1	508	*CACNA1A*	0.10	1
73	*BTN2A1*	0.0096	1	509	*KALRN*	0.10	1
74	*UBE2O*	0.0097	1	510	*CDC25B*	0.10	1
75	*CEP250*	0.010	1	511	*AMBRA1*	0.10	1
76	*MAMDC4*	0.010	1	512	*FSCN2*	0.10	1
77	*ADAMTS9*	0.011	1	513	*MRPL55*	0.10	1
78	*KRT72*	0.011	1	514	*APOB*	0.10	1
79	*RUSC2*	0.011	1	515	*ARHGEF11*	0.10	1
80	*ZNF683*	0.011	1	516	*PRDM15*	0.11	1
81	*SIPA1L3*	0.011	1	517	*COL27A1*	0.11	1
82	*MYOCD*	0.011	1	518	*DUPD1*	0.11	1
83	*DNAH3*	0.011	1	519	*HTR1A*	0.11	1
84	*MUC16*	0.011	1	520	*DOCK5*	0.11	1
85	*SYT12*	0.011	1	521	*ARHGEF18*	0.11	1
86	*CLDN14*	0.011	1	522	*LAMA1*	0.11	1
87	*ATP12A*	0.011	1	523	*HPS6*	0.11	1
88	*DNAH10*	0.011	1	524	*TCTE1*	0.11	1
89	*WDFY4*	0.012	1	525	*CDH24*	0.11	1
90	*ZNF77*	0.012	1	526	*MAML3*	0.11	1
91	*DIS3*	0.012	1	527	*PRR14L*	0.11	1
92	*GADD45GIP1*	0.012	1	528	*PEG3*	0.11	1
93	*ROCK2*	0.012	1	529	*HLA-C*	0.11	1

94	*CSMD2*	0.013	1	530	*TGM4*	0.11	1
95	*AKAP13*	0.013	1	531	*THADA*	0.11	1
96	*WLS*	0.013	1	532	*ZNF423*	0.11	1
97	*SYCP1*	0.013	1	533	*LAMA4*	0.11	1
98	*KIF16B*	0.014	1	534	*TULP4*	0.11	1
99	*SLC12A3*	0.014	1	535	*DPYSL3*	0.11	1
100	*TJP2*	0.014	1	536	*SEC24C*	0.11	1
101	*CROCC*	0.014	1	537	*KIF20B*	0.12	1
102	*RLF*	0.014	1	538	*PCSK9*	0.12	1
103	*GAS2L2*	0.014	1	539	*GAB2*	0.12	1
104	*NGDN*	0.014	1	540	*USP54*	0.12	1
105	*HAL*	0.014	1	541	*MYBPC3*	0.12	1
106	*ENDOU*	0.014	1	542	*FREM2*	0.12	1
107	*BAZ1B*	0.015	1	543	*VAT1L*	0.12	1
108	*ZRANB3*	0.015	1	544	*MGA*	0.12	1
109	*AGBL5*	0.015	1	545	*HELZ*	0.12	1
110	*SEC31B*	0.015	1	546	*KAT6A*	0.12	1
111	*ADAMTS7*	0.015	1	547	*ITSN1*	0.12	1
112	*FPGT*	0.015	1	548	*DCT*	0.12	1
113	*TMPRSS9*	0.016	1	549	*SMG1*	0.12	1
114	*MYOM3*	0.016	1	550	*FBXO40*	0.12	1
115	*EPB41L2*	0.016	1	551	*SYTL2*	0.12	1
116	*LRRIQ1*	0.016	1	552	*KRTAP10-9*	0.13	1
117	*NPHS1*	0.017	1	553	*TRIM47*	0.13	1

118	*FAM91A1*	0.017	1	554	*ABCC2*	0.13	1
119	*SLC7A2*	0.017	1	555	*ABCC11*	0.13	1
120	*MICALL2*	0.018	1	556	*SPATA20*	0.13	1
121	*DNAH17*	0.018	1	557	*TG*	0.13	1
122	*KNG1*	0.018	1	558	*TPR*	0.13	1
123	*DNAH14*	0.018	1	559	*ACSM4*	0.13	1
124	*CILP2*	0.018	1	560	*PDCD11*	0.13	1
125	*HIVEP3*	0.018	1	561	*ATP8B3*	0.13	1
126	*GEMIN5*	0.018	1	562	*DPH1*	0.13	1
127	*ATG16L2*	0.019	1	563	*LAMP3*	0.13	1
128	*MDM1*	0.019	1	564	*PRX*	0.13	1
129	*STARD9*	0.019	1	565	*ZDHHC6*	0.13	1
130	*TRAK1*	0.019	1	566	*ANKRD29*	0.13	1
131	*ASTL*	0.019	1	567	*SLC27A4*	0.13	1
132	*MFSD10*	0.019	1	568	*FSIP2*	0.13	1
133	*MYOM2*	0.019	1	569	*EPN1*	0.13	1
134	*ITGB8*	0.019	1	570	*PIK3C2B*	0.13	1
135	*HES7*	0.019	1	571	*AMZ1*	0.13	1
136	*PYROXD2*	0.020	1	572	*VGLL2*	0.13	1
137	*ZNF629*	0.020	1	573	*BIRC6*	0.14	1
138	*EVPL*	0.020	1	574	*OBSL1*	0.14	1
139	*AP4B1*	0.020	1	575	*KIAA2026*	0.14	1
140	*SLITRK5*	0.020	1	576	*ARHGEF17*	0.14	1
141	*ZNF839*	0.020	1	577	*OR7G1*	0.14	1

142	*DLG5*	0.020	1	578	*SCN9A*	0.14	1
143	*RRN3*	0.020	1	579	*SNX21*	0.14	1
144	*CARD17*	0.020	1	580	*EPS8L3*	0.14	1
145	*PCLO*	0.021	1	581	*PLB1*	0.14	1
146	*COL24A1*	0.021	1	582	*LAMB2*	0.14	1
147	*TNRC6C*	0.021	1	583	*MUC6*	0.14	1
148	*UPK2*	0.021	1	584	*VWF*	0.14	1
149	*CACNA1D*	0.021	1	585	*ZNF726*	0.14	1
150	*LRP2*	0.021	1	586	*DMBT1*	0.14	1
151	*PFAS*	0.021	1	587	*HSPG2*	0.15	1
152	*EGFR*	0.021	1	588	*FHIT*	0.15	1
153	*CELSR2*	0.021	1	589	*BACH2*	0.15	1
154	*CARD11*	0.022	1	590	*ZNF737*	0.15	1
155	*EXT2*	0.022	1	591	*ASB14*	0.15	1
156	*LRPPRC*	0.022	1	592	*ZNF391*	0.15	1
157	*RIC8A*	0.022	1	593	*CROT*	0.15	1
158	*EMILIN1*	0.022	1	594	*HPGDS*	0.15	1
159	*FLNB*	0.022	1	595	*ATMIN*	0.15	1
160	*FBXL6*	0.022	1	596	*MFAP3*	0.15	1
161	*HIST1H3A*	0.023	1	597	*COL2A1*	0.15	1
162	*ARHGEF40*	0.023	1	598	*CC2D2A*	0.15	1
163	*CEP290*	0.023	1	599	*SEZ6L2*	0.15	1
164	*ZMYM5*	0.023	1	600	*TRIM65*	0.15	1
165	*DPY19L1*	0.023	1	601	*MRC2*	0.15	1

166	*PPAN*	0.023	1	602	*DNAH2*	0.15	1
167	*PPAN-P2RY11*	0.023	1	603	*POLN*	0.15	1
168	*NSUN5*	0.023	1	604	*KDM6B*	0.15	1
169	*ZMYM6*	0.023	1	605	*NUP98*	0.15	1
170	*CSPG4*	0.024	1	606	*FER1L5*	0.15	1
171	*DARS2*	0.024	1	607	*PPFIA2*	0.15	1
172	*COL6A6*	0.024	1	608	*MYLK*	0.16	1
173	*ANGEL2*	0.024	1	609	*TNN*	0.16	1
174	*TGM1*	0.025	1	610	*NPC1L1*	0.16	1
175	*CORO7-PAM16*	0.025	1	611	*MPHOSPH10*	0.16	1
176	*SDK2*	0.025	1	612	*ACACB*	0.16	1
177	*CNTN5*	0.025	1	613	*NOC4L*	0.16	1
178	*LCTL*	0.025	1	614	*KRT73*	0.16	1
179	*BTBD11*	0.025	1	615	*APLP2*	0.16	1
180	*CORO7*	0.025	1	616	*SCN10A*	0.16	1
181	*TOP1MT*	0.025	1	617	*EVC2*	0.16	1
182	*CAPN10*	0.025	1	618	*WDR20*	0.16	1
183	*MICAL3*	0.025	1	619	*CILP*	0.16	1
184	*JPH3*	0.025	1	620	*TBKBP1*	0.16	1
185	*ANKRD6*	0.026	1	621	*PPL*	0.16	1
186	*ARRDC4*	0.026	1	622	*MYH1*	0.16	1
187	*ABCC8*	0.026	1	623	*SCARF1*	0.16	1
188	*KNTC1*	0.026	1	624	*HPS3*	0.16	1
189	*CPSF1*	0.026	1	625	*KYNU*	0.16	1

190	*KIF21A*	0.026	1	626	*XPO7*	0.16	1
191	*CDHR1*	0.027	1	627	*RALGAPA2*	0.16	1
192	*SYTL3*	0.027	1	628	*FCHO1*	0.16	1
193	*MYO7A*	0.027	1	629	*TCF7*	0.16	1
194	*BCAM*	0.027	1	630	*ABCC6*	0.16	1
195	*REV3L*	0.027	1	631	*PRUNE2*	0.17	1
196	*LIMD1*	0.028	1	632	*MYOF*	0.17	1
197	*VPS13D*	0.028	1	633	*ABCA2*	0.17	1
198	*UNC13B*	0.028	1	634	*ARHGAP33*	0.17	1
199	*TIMD4*	0.028	1	635	*RECQL4*	0.17	1
200	*SHANK1*	0.028	1	636	*RBM34*	0.17	1
201	*UBAP2*	0.029	1	637	*UGGT1*	0.17	1
202	*GLIS3*	0.029	1	638	*GMPR*	0.17	1
203	*SSC5D*	0.029	1	639	*FGGY*	0.17	1
204	*CHRM2*	0.029	1	640	*SCAF4*	0.17	1
205	*PKHD1*	0.029	1	641	*PP2D1*	0.17	1
206	*TMEM143*	0.030	1	642	*COBLL1*	0.17	1
207	*HELLS*	0.030	1	643	*SIPA1L2*	0.17	1
208	*HPR*	0.030	1	644	*TMPRSS7*	0.17	1
209	*MMRN2*	0.030	1	645	*ERCC6*	0.17	1
210	*LRP4*	0.030	1	646	*ZFPM1*	0.18	1
211	*CLK3*	0.030	1	647	*PCNT*	0.18	1
212	*MACF1*	0.030	1	648	*PLCE1*	0.18	1
213	*MYO9A*	0.030	1	649	*DMXL1*	0.18	1

214	*SERPINF1*	0.031	1	650	*DAAM2*	0.18	1
215	*ZAR1*	0.031	1	651	*SNX9*	0.18	1
216	*TTC37*	0.031	1	652	*MMP14*	0.18	1
217	*ERCC5*	0.031	1	653	*FASN*	0.18	1
218	*CDH1*	0.031	1	654	*AGTPBP1*	0.18	1
219	*BIVM-ERCC5*	0.031	1	655	*DSCAM*	0.18	1
220	*SCN11A*	0.031	1	656	*C1orf127*	0.18	1
221	*MS4A18*	0.032	1	657	*PLA2G4B*	0.18	1
222	*MAN2A1*	0.032	1	658	*KLK10*	0.18	1
223	*PLXNA4*	0.032	1	659	*DNAH8*	0.18	1
224	*TMEM139*	0.033	1	660	*KRTAP5-6*	0.18	1
225	*TNRC18*	0.033	1	661	*RHBDF2*	0.18	1
226	*KRT17*	0.033	1	662	*ZSWIM4*	0.18	1
227	*KIF12*	0.033	1	663	*ROR2*	0.18	1
228	*MIIP*	0.033	1	664	*CHTF18*	0.18	1
229	*AP1G2*	0.034	1	665	*BAZ2A*	0.19	1
230	*NEFM*	0.034	1	666	*GPR162*	0.19	1
231	*CYP2A13*	0.034	1	667	*LIPJ*	0.19	1
232	*PKD2*	0.034	1	668	*TROAP*	0.19	1
233	*ASPM*	0.034	1	669	*ZNF879*	0.19	1
234	*CMA1*	0.034	1	670	*PLCB2*	0.19	1
235	*MAP1B*	0.034	1	671	*BCHE*	0.19	1
236	*LRCH3*	0.034	1	672	*TMEM43*	0.19	1
237	*DISP1*	0.035	1	673	*POGLUT1*	0.19	1

238	*UHRF1BP1*	0.035	1	674	*MANEAL*	0.19	1
239	*KANK1*	0.035	1	675	*EME2*	0.20	1
240	*CDKL4*	0.035	1	676	*RYR1*	0.20	1
241	*UNC45B*	0.036	1	677	*EXD2*	0.20	1
242	*NTNG1*	0.036	1	678	*CAPN13*	0.20	1
243	*FILIP1L*	0.036	1	679	*FAM83E*	0.20	1
244	*FZD6*	0.036	1	680	*PRRC2A*	0.20	1
245	*ZNF318*	0.037	1	681	*GOLGA5*	0.20	1
246	*ZNF845*	0.037	1	682	*MYO18A*	0.20	1
247	*ROBO3*	0.037	1	683	*DUSP12*	0.20	1
248	*MTMR11*	0.037	1	684	*HIP1R*	0.20	1
249	*HIPK4*	0.037	1	685	*JMJD7-PLA2G4B*	0.20	1
250	*SBF2*	0.038	1	686	*ANKLE2*	0.21	1
251	*ROS1*	0.038	1	687	*NOTCH4*	0.21	1
252	*RNF43*	0.038	1	688	*SLCO1A2*	0.21	1
253	*SETD6*	0.038	1	689	*SMPD3*	0.21	1
254	*FLNC*	0.039	1	690	*ABCA13*	0.21	1
255	*EDEM3*	0.039	1	691	*CCDC40*	0.21	1
256	*LARS*	0.039	1	692	*CHCHD5*	0.21	1
257	*RPS6KB2*	0.039	1	693	*ITGB4*	0.21	1
258	*HR*	0.040	1	694	*DNAH1*	0.21	1
259	*UTRN*	0.040	1	695	*F3*	0.21	1
260	*SH3TC1*	0.040	1	696	*PKD1*	0.21	1
261	*CAMTA1*	0.040	1	697	*TNC*	0.22	1

262	*LRRIQ3*	0.040	1	698	*SPTAN1*	0.22	1
263	*NFX1*	0.041	1	699	*ZNF525*	0.22	1
264	*EPRS*	0.041	1	700	*HLA-F*	0.22	1
265	*GSDMA*	0.041	1	701	*POSTN*	0.22	1
266	*CACNA1S*	0.041	1	702	*OVCH1*	0.22	1
267	*PCDHGB6*	0.041	1	703	*ZNF729*	0.22	1
268	*MAP1S*	0.041	1	704	*FUT5*	0.22	1
269	*TDRD6*	0.041	1	705	*AUTS2*	0.22	1
270	*PIEZO1*	0.042	1	706	*CTSB*	0.22	1
271	*PPIC*	0.042	1	707	*THSD1*	0.22	1
272	*MYO15A*	0.042	1	708	*GNA15*	0.22	1
273	*OTOG*	0.042	1	709	*CASP14*	0.22	1
274	*ABCA8*	0.042	1	710	*UBA7*	0.23	1
275	*SPTBN5*	0.042	1	711	*CAND2*	0.23	1
276	*IQGAP1*	0.042	1	712	*LTB4R2*	0.23	1
277	*GALC*	0.042	1	713	*PRDM10*	0.23	1
278	*WDR11*	0.043	1	714	*ZNF16*	0.23	1
279	*NCKAP5*	0.043	1	715	*ROBO1*	0.23	1
280	*TLN1*	0.043	1	716	*TGFBR2*	0.23	1
281	*ABI3BP*	0.043	1	717	*DCC*	0.24	1
282	*ZSCAN29*	0.043	1	718	*DQX1*	0.24	1
283	*SETMAR*	0.044	1	719	*WDR90*	0.24	1
284	*ERBB3*	0.044	1	720	*OR6S1*	0.24	1
285	*SLC5A4*	0.045	1	721	*EML3*	0.24	1

286	*KNDC1*	0.045	1	722	*STAB2*	0.24	1
287	*UBR1*	0.045	1	723	*C14orf180*	0.24	1
288	*CNGB3*	0.045	1	724	*BNC1*	0.24	1
289	*CNR1*	0.045	1	725	*CCDC66*	0.24	1
290	*AHNAK2*	0.045	1	726	*ACVR1C*	0.24	1
291	*ENTPD1*	0.046	1	727	*SIGLEC11*	0.24	1
292	*CADPS*	0.046	1	728	*DCBLD1*	0.25	1
293	*ESYT2*	0.046	1	729	*MYOG*	0.25	1
294	*MTPAP*	0.046	1	730	*SNAPC4*	0.25	1
295	*SHANK2*	0.046	1	731	*SRSF4*	0.25	1
296	*CHST12*	0.047	1	732	*MYH7B*	0.25	1
297	*ADAM7*	0.047	1	733	*ANKRD31*	0.25	1
298	*NEDD4*	0.047	1	734	*SLCO6A1*	0.25	1
299	*RYR2*	0.048	1	735	*HIVEP1*	0.25	1
300	*ABCA4*	0.048	1	736	*SKA3*	0.26	1
301	*ASTN2*	0.049	1	737	*GFM2*	0.26	1
302	*CDAN1*	0.050	1	738	*TNXB*	0.26	1
303	*PCDH8*	0.050	1	739	*OR5K3*	0.26	1
304	*MYH9*	0.050	1	740	*PRPH*	0.26	1
305	*FCN1*	0.050	1	741	*STAB1*	0.26	1
306	*GPATCH8*	0.050	1	742	*GRIN3B*	0.26	1
307	*COL20A1*	0.050	1	743	*NUP214*	0.27	1
308	*FAT3*	0.050	1	744	*KIF26A*	0.28	1
309	*MCTP2*	0.051	1	745	*NOP14*	0.28	1

310	*ZNF99*	0.051	1	746	*PTGIS*	0.28	1
311	*TXNDC2*	0.051	1	747	*CHMP7*	0.28	1
312	*C4orf47*	0.052	1	748	*DST*	0.28	1
313	*GLIS1*	0.052	1	749	*SPTA1*	0.28	1
314	*DSPP*	0.053	1	750	*NDST4*	0.29	1
315	*PIGS*	0.053	1	751	*ANKAR*	0.29	1
316	*RSF1*	0.053	1	752	*RAPGEF4*	0.29	1
317	*SRRM2*	0.053	1	753	*CUL9*	0.29	1
318	*SUSD2*	0.053	1	754	*CD109*	0.29	1
319	*PTPN13*	0.054	1	755	*B3GALT2*	0.29	1
320	*APIP*	0.054	1	756	*CLRN1*	0.29	1
321	*PCDH1*	0.054	1	757	*ARMC2*	0.29	1
322	*MOAP1*	0.054	1	758	*SMAP1*	0.29	1
323	*PKD2L1*	0.054	1	759	*LRRC15*	0.29	1
324	*RBM28*	0.054	1	760	*INSR*	0.29	1
325	*TTC24*	0.054	1	761	*C10orf88*	0.30	1
326	*SYTL1*	0.055	1	762	*SEC24D*	0.30	1
327	*CASC3*	0.055	1	763	*STON1-GTF2A1L*	0.30	1
328	*TTC3*	0.055	1	764	*ATP10A*	0.30	1
329	*RASA3*	0.056	1	765	*ITGAM*	0.30	1
330	*OR4E1*	0.056	1	766	*TBL3*	0.30	1
331	*DPP7*	0.056	1	767	*ZNF572*	0.31	1
332	*MAN2A2*	0.057	1	768	*SCARA3*	0.31	1
333	*ZNF469*	0.057	1	769	*PCDHA4*	0.31	1

334	*MAP3K8*	0.057	1	770	*UGT1A4*	0.32	1
335	*COL6A2*	0.057	1	771	*WFS1*	0.32	1
336	*DNMBP*	0.057	1	772	*HAUS6*	0.32	1
337	*SIGLEC15*	0.058	1	773	*MEGF8*	0.33	1
338	*FRZB*	0.058	1	774	*BPIFC*	0.33	1
339	*SIRT3*	0.058	1	775	*ELMO1*	0.33	1
340	*SLC1A7*	0.058	1	776	*IGSF10*	0.33	1
341	*ABCC3*	0.058	1	777	*ZNF408*	0.33	1
342	*DCHS1*	0.058	1	778	*NID1*	0.34	1
343	*IL11RA*	0.058	1	779	*AHNAK*	0.34	1
344	*KIF4B*	0.059	1	780	*ABCA10*	0.34	1
345	*SMTN*	0.059	1	781	*TAF1L*	0.34	1
346	*LCOR*	0.059	1	782	*ABCB5*	0.34	1
347	*TEKT3*	0.060	1	783	*DDX55*	0.35	1
348	*ZFYVE16*	0.060	1	784	*PCDHGA5*	0.35	1
349	*OR8K5*	0.060	1	785	*USP43*	0.36	1
350	*LRP1*	0.061	1	786	*PDZD2*	0.36	1
351	*MYH8*	0.061	1	787	*EPB41*	0.36	1
352	*SLC27A3*	0.061	1	788	*FREM1*	0.36	1
353	*DSP*	0.061	1	789	*PCDHA8*	0.38	1
354	*DENND2C*	0.062	1	790	*PDLIM2*	0.38	1
355	*RBM15B*	0.062	1	791	*IGDCC3*	0.38	1
356	*SPG11*	0.062	1	792	*MORN1*	0.39	1
357	*PABPN1L*	0.063	1	793	*COL6A3*	0.39	1

358	*TEKT2*	0.063	1	794	*OR4A15*	0.39	1
359	*TMPRSS5*	0.063	1	795	*SEMA4G*	0.40	1
360	*ALPK3*	0.063	1	796	*PLEKHA6*	0.40	1
361	*VCAN*	0.063	1	797	*KIRREL2*	0.40	1
362	*PLEC*	0.064	1	798	*HRNR*	0.41	1
363	*TULP2*	0.064	1	799	*ZP4*	0.41	1
364	*EPPK1*	0.064	1	800	*KARS*	0.42	1
365	*FOXS1*	0.064	1	801	*ATP10B*	0.42	1
366	*CCDC158*	0.064	1	802	*LRRC46*	0.42	1
367	*CABIN1*	0.064	1	803	*LRRC66*	0.42	1
368	*STXBP5L*	0.065	1	804	*ZNF880*	0.43	1
369	*TNK2*	0.065	1	805	*CELSR3*	0.43	1
370	*MCM3AP*	0.065	1	806	*BRSK2*	0.43	1
371	*FAM20C*	0.065	1	807	*ABCC4*	0.44	1
372	*NLRP14*	0.066	1	808	*MPL*	0.45	1
373	*TUBGCP6*	0.066	1	809	*PRRC2B*	0.45	1
374	*LTK*	0.066	1	810	*GCKR*	0.46	1
375	*OR4B1*	0.066	1	811	*BFSP2*	0.47	1
376	*ZNF571*	0.067	1	812	*CUBN*	0.48	1
377	*NFE2L3*	0.068	1	813	*DGCR8*	0.50	1
378	*MIA2*	0.069	1	814	*ZNF792*	0.50	1
379	*FBN3*	0.069	1	815	*PPP2R3A*	0.50	1
380	*HSF5*	0.069	1	816	*SEMA5B*	0.53	1
381	*RAPH1*	0.069	1	817	*HMCN1*	0.54	1

382	*FIGN*	0.069	1	818	*IRX4*	0.54	1
383	*BRD8*	0.070	1	819	*PTCH1*	0.54	1
384	*ZDBF2*	0.070	1	820	*DICER1*	0.54	1
385	*CSPP1*	0.070	1	821	*MSH4*	0.55	1
386	*ZNF333*	0.070	1	822	*CASZ1*	0.57	1
387	*EVC*	0.070	1	823	*PRODH*	0.59	1
388	*OR6P1*	0.070	1	824	*SLIT3*	0.59	1
389	*PLIN4*	0.070	1	825	*PKHD1L1*	0.61	1
390	*SIGLEC10*	0.070	1	826	*DCDC1*	0.61	1
391	*DZIP1*	0.071	1	827	*SPHKAP*	0.67	1
392	*NRXN1*	0.071	1	828	*ENAM*	0.72	1
393	*CGN*	0.071	1	829	*COL28A1*	1	1
394	*PTK6*	0.071	1	830	*CPNE5*	1	1
395	*RNPEPL1*	0.072	1	831	*MCM8*	1	1
396	*RGS14*	0.072	1	832	*FADS6*	1	1
397	*PPFIA4*	0.072	1	833	*CYP2A7*	1	1
398	*UNC13D*	0.072	1	834	*PLCG1*	1	1
399	*FAM131C*	0.073	1	835	*RNF17*	1	1
400	*KRI1*	0.073	1	836	*CNTN6*	1	1
401	*KEL*	0.073	1	837	*DYSF*	1	1
402	*AGL*	0.073	1	838	*LYAR*	1	1
403	*POPDC2*	0.073	1	839	*ADAMTSL4*	1	1
404	*CEP19*	0.074	1	840	*FZD4*	1	1
405	*UBR4*	0.074	1	841	*COL9A2*	1	1

406	*KIF7*	0.074	1	842	*MAP1A*	1	1
407	*KBTBD13*	0.074	1	843	*MLLT10*	1	1
408	*MYO18B*	0.074	1	844	*HLA-DMA*	1	1
409	*UTP3*	0.075	1	845	*PROM1*	1	1
410	*NUP188*	0.075	1	846	*MUC17*	1	1
411	*SYNJ2*	0.075	1	847	*RTN1*	1	1
412	*ZNF862*	0.075	1	848	*LAMA2*	1	1
413	*FRYL*	0.075	1	849	*NEBL*	1	1
414	*ZFYVE19*	0.076	1	850	*ADCY10*	1	1
415	*ABCF3*	0.076	1	851	*LILRB2*	1	1
416	*SPTBN2*	0.076	1	852	*MEA1*	1	1
417	*DDI2*	0.077	1	853	*ST18*	1	1
418	*ACO1*	0.077	1	854	*MYO3B*	1	1
419	*SLC9A9*	0.077	1	855	*ADIPOQ*	1	1
420	*OPLAH*	0.077	1	856	*C4orf50*	1	1
421	*SYNE1*	0.077	1	857	*COL4A2*	1	1
422	*SLC5A6*	0.078	1	858	*SART1*	1	1
423	*LOXHD1*	0.079	1	859	*PTGIR*	1	1
424	*GBF1*	0.080	1	860	*TMEM104*	1	1
425	*LRP3*	0.080	1	861	*ARID3C*	1	1
426	*FOPNL*	0.080	1	862	*LRRC39*	1	1
427	*PNPLA7*	0.080	1	863	*TXLNB*	1	1
428	*ITIH3*	0.080	1	864	*ZNF534*	1	1
429	*SORCS2*	0.080	1	865	*CACNA1C*	1	1

430	*MCM5*	0.081	1	866	*C7orf31*	1	1
431	*JAG1*	0.081	1	867	*CMYA5*	1	1
432	*PACSIN2*	0.081	1	868	*SH3D21*	1	1
433	*RREB1*	0.082	1	869	*CAPRIN2*	1	1
434	*TMCO3*	0.082	1	870	*CPZ*	1	1
435	*ATP10D*	0.082	1	871	*TMEM54*	1	1
436	*PIWIL2*	0.082	1	872	*URB2*	1	1

*: Bonferroni correction

Table S3. Four core features and two indicative biomarkers of 45 DLB patients

Criteria	Features				Indicative biomarkers		# subjects
	F1	F2	F3	F4	MIBG	SPECT	
				✓	✓		1
				✓		✓	1
			✓		✓		3
One feature			✓			✓	7
		✓			✓		8
		✓				✓	4
	✓				✓		1
			✓	✓	✓		1
		✓		✓		✓	2
		✓	✓		✓		3
		✓	✓			✓	1
Two features	✓			✓		✓	1
	✓	✓			✓		5
	✓	✓				✓	2
	✓		✓			✓	1
		✓	✓	✓	✓		1
Three features	✓		✓	✓		✓	2
	✓	✓	✓		✓		1
Total	13	27	20	9	24	21	45

F1, cognitive fluctuation; F2, visual hallucinations; F3, parkinsonism; F4, REM sleep behavior disorder

Abbreviations: MIBG, metaiodobenzylguanidine myocardial scintigraphy; SPECT, single-photon emission computed tomography

Table S4. Four core features of 8 DLB patients with *CDH23* variants

SNP number	Sample ID	F1	F2	F3	F4	Subjective hearing loss
rs181275139	DLB01	✓	✓	✓		yes
	DLB02	✓	✓			yes
rs563688802	DLB03		✓			yes
	DLB04		✓			yes
rs137937502	DLB05	✓	✓			yes
	DLB06			✓		yes
	DLB07			✓		yes
	DLB08				✓	NA

F1, cognitive fluctuation; F2, visual hallucinations; F3, parkinsonism; F4, REM sleep behavior disorder
